# Intracranial Hypotension in the Setting of Post-Concussion Headache: A Case Series

**DOI:** 10.7759/cureus.10526

**Published:** 2020-09-18

**Authors:** Eric W Pettyjohn, Robert M Donlan, John Breck, James R Clugston

**Affiliations:** 1 Family Medicine, University of Florida Health, Gainesville, USA; 2 Sports Medicine, DCH Center for Occupational Health, Tuscaloosa, USA; 3 Sports Medicine, University of Colorado, Boulder, USA; 4 Sports Medicine, University of Florida Health, Gainesville, USA

**Keywords:** intracranial hypotension, csf leak, csf hypovolemia, concussion, post-concussion headache, american football, post-traumatic headache, autologous blood patch

## Abstract

Intracranial hypotension can be a common sequela of a cerebrospinal fluid (CSF) leak. However, evidence of such a condition related to an injury in American football is currently lacking in the literature. While a positional or orthostatic headache is the most classic symptom of headaches due to intracranial hypotension, a variety of nonspecific symptoms such as neck pain, nausea, vomiting, photophobia, phonophobia, and visual changes can also be present. We present two cases where collegiate American football players developed protracted headaches after a concussive injury and were subsequently diagnosed with intracranial hypotension thought secondary to spinal CSF leaks.

Both players underwent multiple procedures of fluoroscopic-guided autologous blood patching, with improvement in their headaches. Recovery varied between the athletes. Case 1 achieved full resolution of his headaches and returned to full activity. Case 2 continued to have intermittent headaches after blood patching, but the positional nature had resolved and he was cleared for full participation in football and was closely followed during the remainder of his collegiate career. Both these cases emphasize the importance of including CSF leak as a cause of post-traumatic headache in an American football player.

## Introduction

Cerebrospinal fluid (CSF) mechanically supports the central nervous system (CNS) and influences CNS physiologic function [1]. As a fluid in a confined space, CSF volume contributes to intracranial pressure and changes in this pressure can cause a variety of symptoms and pathologies. Decreased intracranial pressure can lead to intracranial hypotension, which has been associated with a postural headache, described as diffuse head pain worse with standing, sitting, or head motion and improved with lying supine [2].

To our knowledge, intracranial hypotension has not been described following an injury in American football, and has also not been acknowledged as a possible cause for prolonged concussion symptoms. In this series, we present two cases from collegiate American football where players sustained clinically diagnosed concussions with prolonged headache and were subsequently found to have clinical and imaging findings of intracranial hypotension thought secondary to spinal CSF leaks. Both patients had significant cervical motion (hyperextension) during their injuries.

## Case presentation

Case 1

A healthy 20-year-old male American football player jumped to catch a ball and landed on his back, leading to a sudden hyperextension of his cervical spine before subsequent contact of the posterior portion of his helmet with the ground. He did not report any symptoms at the time and played the remainder of the game. The next day, he presented to the athletic training facility complaining of headache, dizziness, neck spasms, and insomnia. Further questioning revealed amnesia of the events directly before and after the injury and the trip home. He denied any nausea, vomiting, or visual changes. He had one previous concussion 18 months prior with symptom resolution in one week. Personal and family histories were negative for migraine or other persistent headaches. He denied medication use.

On physical examination, he was in no acute distress, was alert and oriented to year, month, and day of the week, but not date. Neurological examination, including cranial nerves 2-12, cerebellar testing with finger-to-nose and heel-to-toe walking, Romberg maneuver, and a subjective simple reaction time maneuver, was normal. Neck examination demonstrated full range of motion (ROM) in all planes with mild subjective paraspinal stiffness bilaterally but no midline tenderness. He had normal strength and sensation in all four extremities. He had no tenderness with palpation of the head, including the occipital area. Post Concussion Symptom Scale (PCSS) was >0 in 15 of 22 symptoms with a severity of 42 (prior baseline was >0 for 3 symptoms with a severity of 7), and Standardized Assessment for Concussion (SAC) was 24/30 with a delayed recall subscore of 2/5 compared to his baseline of 26/30 also with a delayed recall of 2/5. Balance Error Scoring System (BESS) was not performed. Computer-based neurocognitive evaluation with the Immediate Post-concussion Assessment and Cognitive Testing (ImPACT™) battery did not exceed the Reliable Change Index in any category compared to his baseline and trended toward improvement in memory composite visual, visual motor speed, and reaction time.

At this time, he was diagnosed with a concussion and a cervical strain. He was treated initially with rest, including exclusion from practice, team meetings, and classes. PCSS, SAC, and neck stiffness returned to normal after two days. He was advanced to football activity including contact practice by day 7.

He reported no problems and appeared to be doing well until 7.5 weeks after his original injury when he pulled himself out of practice with a severe headache. He revealed that he had been having these headaches intermittently since returning from the concussion but had not reported them. He said he had felt tightness in his neck and shoulders and had been receiving neck massages after practice which seemed to help. He noticed the headache with most head movements during activities including weightlifting, running, and even brushing his teeth. Though his neurological examination remained normal, a head CT scan was obtained which was read as normal. He was diagnosed with tension headaches, started on acetaminophen and a nonsteroidal anti-inflammatory drug (NSAID) as needed, and returned to play. He did not report any problems for the remainder of the season.

**Figure 1:**
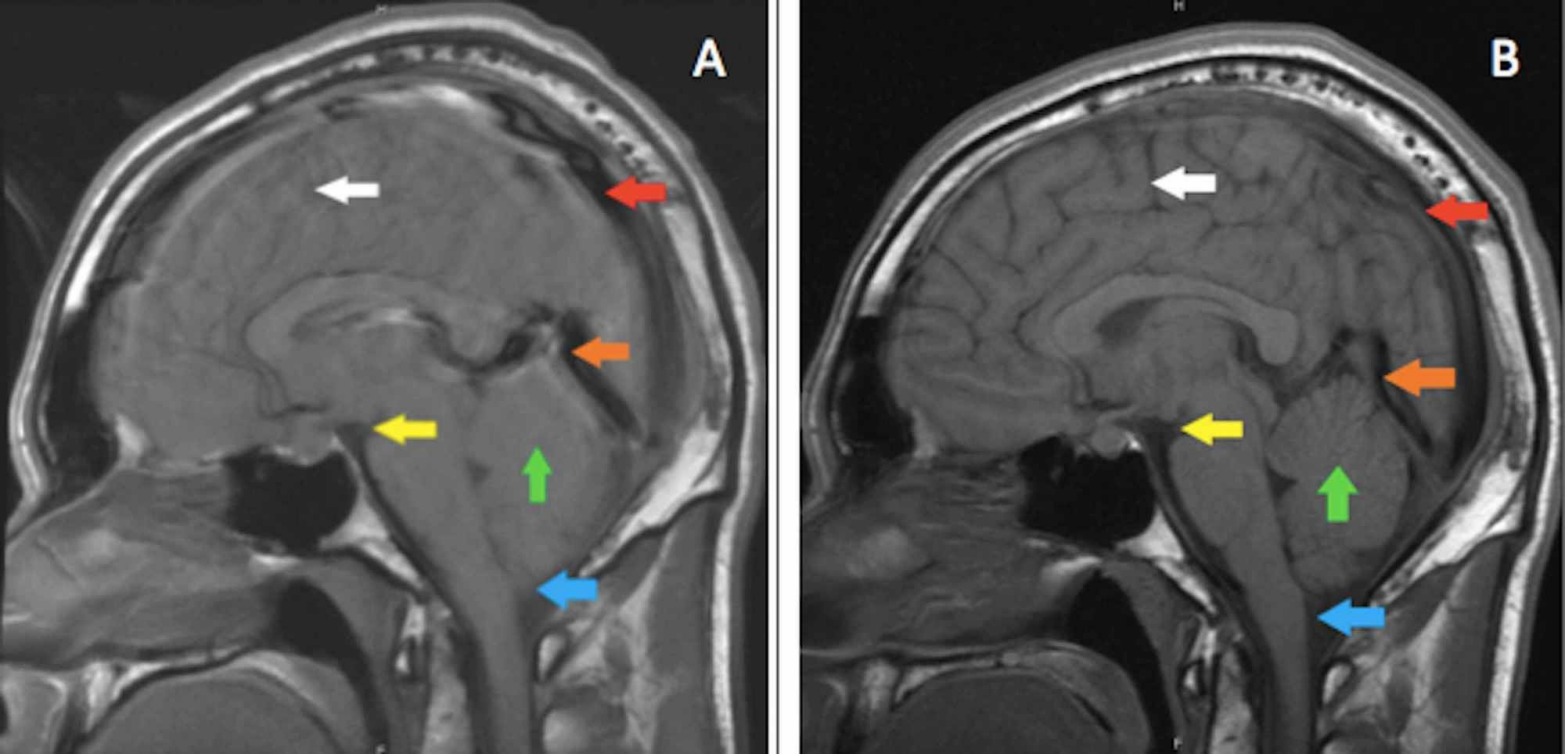
Brain MRI with findings of intracranial hypotension (A) Initial brain MRI with findings of intracranial hypotension prior to blood patching. Significant findings include compression of cortical gyri (white arrow), increased space inside the cavity (red arrow), venous distension (orange arrow), decreased mamillopontine distance (yellow arrow), compression of the cerebellar gyri (green arrow), and a "low riding" brainstem at the foramen magnum (blue arrow). (B) Image shows improvement of the aforementioned findings at week 34 of injury, after blood patch treatments.

At 20.5 weeks from the original injury (now in spring practice), he presented to the athletic training facility complaining of headaches, again associated with head movement. He stated that the headache improved when he laid down. He admitted to taking acetaminophen/caffeine and NSAIDs daily with only mild relief. Due to his frequent usage, these medications were stopped. He was referred to neurology for further evaluation and a brain MRI was ordered and obtained at week 22. The MRI revealed findings consistent with moderate intracranial hypotension (Figure 1A) including a transverse sinus thrombosis with collateral circulation. 

**Figure 2:**
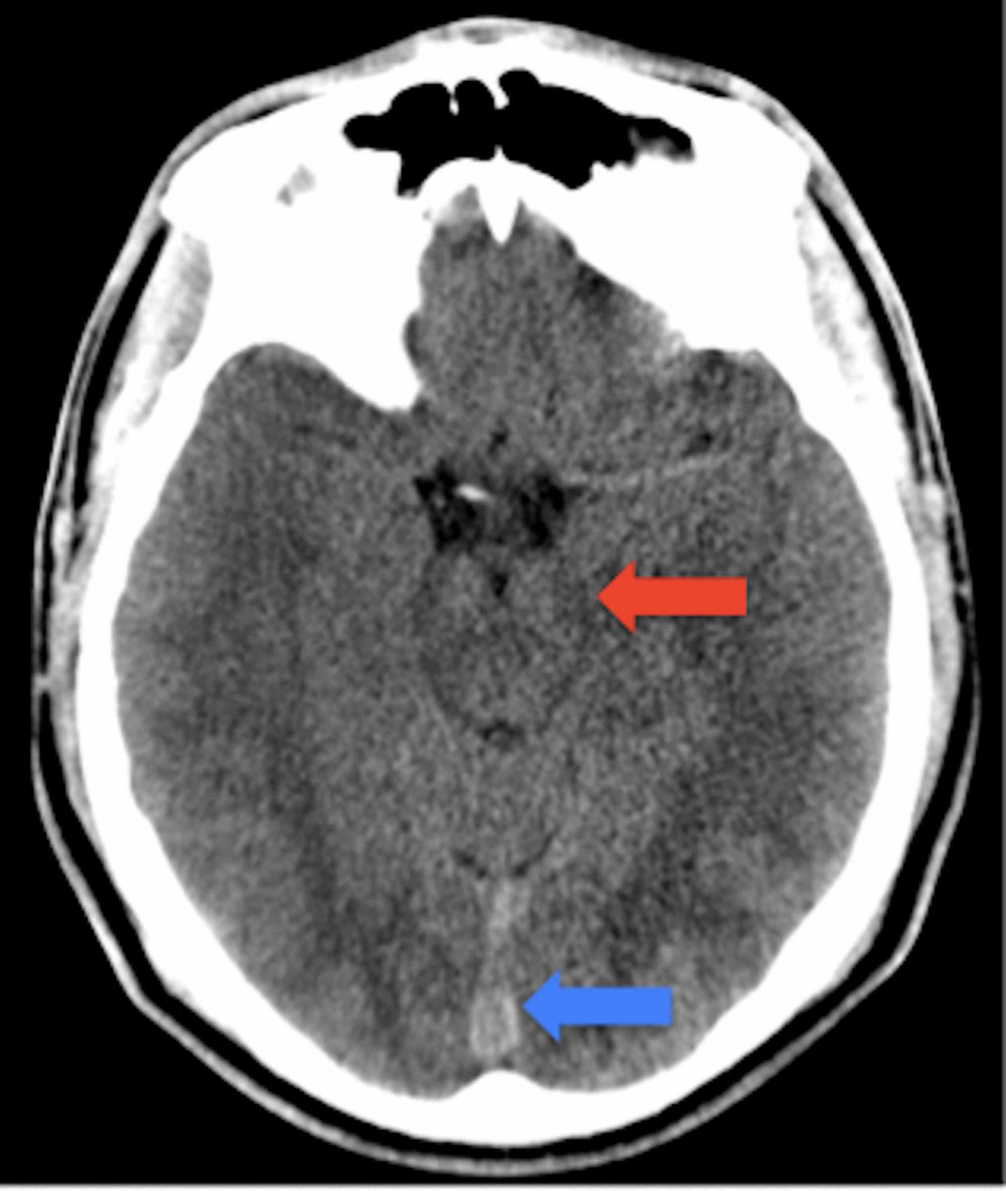
Noncontrast axial CT scan of the head at the level of the suprasellar cistern Mild effacement of crural cistern (red arrow), Prominence of straight sinus (blue arrow).

The prior CT from week 7.5 was also re-evaluated and findings of hypotension were also recognized (Figure 2).

**Figure 3:**
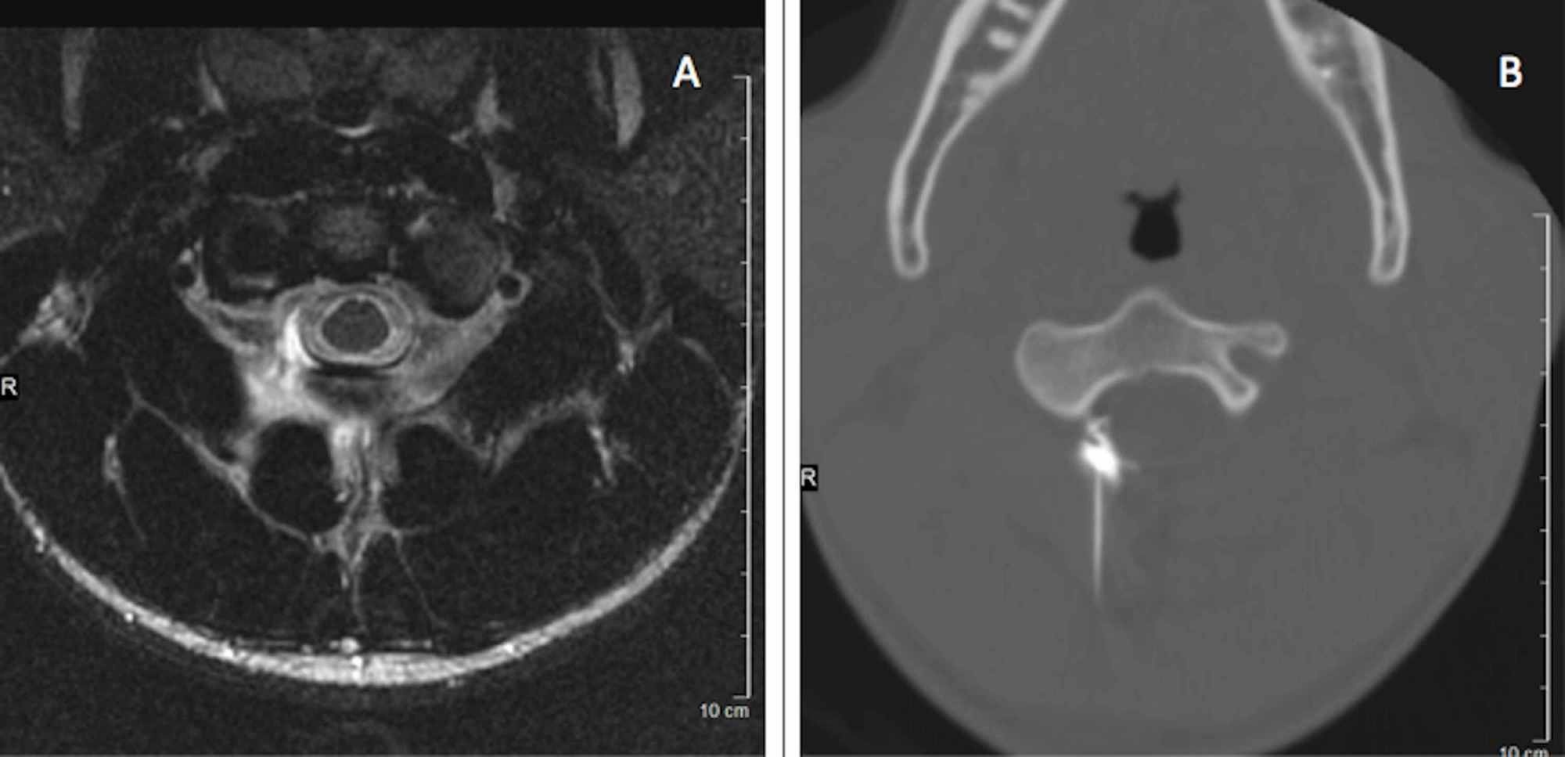
Magnetic resonance (MR) myelogram and CT-guided blood patch (A) T2-weighted MR myelogram image revealing a cervical cerebrospinal fluid leak. (B) CT-guided cervical blood patch targeted at the area of leak seen on MR myelogram.

Given these findings, a CSF leak was considered and a magnetic resonance (MR) myelogram of the cervical, thoracic, and lumbar spine was ordered and obtained at 24 weeks. The study showed evidence of a CSF leak at the C1-C2 level (Figure 3). Since the sinus thrombus seen on the brain MRI at week 22 was felt to be a result of hypovolemia and hypercoagulation labs were normal, anticoagulation medication was not recommended.

The patient was started on caffeine 300 mg orally three times a day as a treatment for the CSF leak. He had no improvement after four days (end of week 24), and a CT-guided 3 ml autologous epidural blood patch (EBP) was then performed at C1-C2 in an attempt to stop the leak (Figure 3B). The patient’s headache improved, and repeat MRI of head and cervical spine at 25 weeks revealed decreased signs of intracranial hypotension including recanalization of the transverse sinus and the suspected site of CSF leak at C1-2 was no longer seen, suggesting the intracranial hypotension had improved. 

At 29 weeks, the headaches, although improved, were not completely resolved. A repeat MRI of his brain, cervical spine, and thoracic spine revealed stable intracranial hypotension, re-thrombosis of the left transverse sinus but no evidence of a cervical or thoracic CSF leak.

At week 31, a multi-level, large volume fluoroscopic-guided autologous blood patch was performed at L2-L3 (12 ml), L3-L4 (12 ml), and L4-L5 (6 ml). By week 32, he was clinically improved and a repeat brain MRI showed mild improvement in intracranial hypotension and partial recanalization of the left transverse sinus. The following day, an additional multi-level, large volume fluoroscopic-guided autologous blood patch was performed at L1-L2 (12 ml) and L5-S1 (12 ml).

By week 34, his headaches were completely resolved and MRI of his brain and heavily weighted T2 non-contrast MRI of his cervical, thoracic, and lumbar spines revealed marked improvement in hypotension with complete resolution of the sinus thrombosis (Figure 1B) and no further CSF leaks. 

He continued to do well and by week 38 was returned to full activity. He was followed closely by team physicians and neurology during the remainder of his collegiate career and continued to play football with no return of headache or any additional concussion like symptoms. 

Case 2

While participating in kickoff coverage, a healthy 21-year-old American football player was hit on the chin, forcing his neck into hyperextension. He did not lose consciousness and ran back to the sideline where he complained of neck pain, dizziness, headache, blurry vision, and amnesia of the hit. He denied any numbness, tingling, or weakness. He was placed in a cervical spine immobilization collar. X-rays of his cervical spine were obtained immediately at the venue and were normal. At that time, he was diagnosed with a concussion and cervical muscle strain and was held out of all football activities.

**Figure 4:**
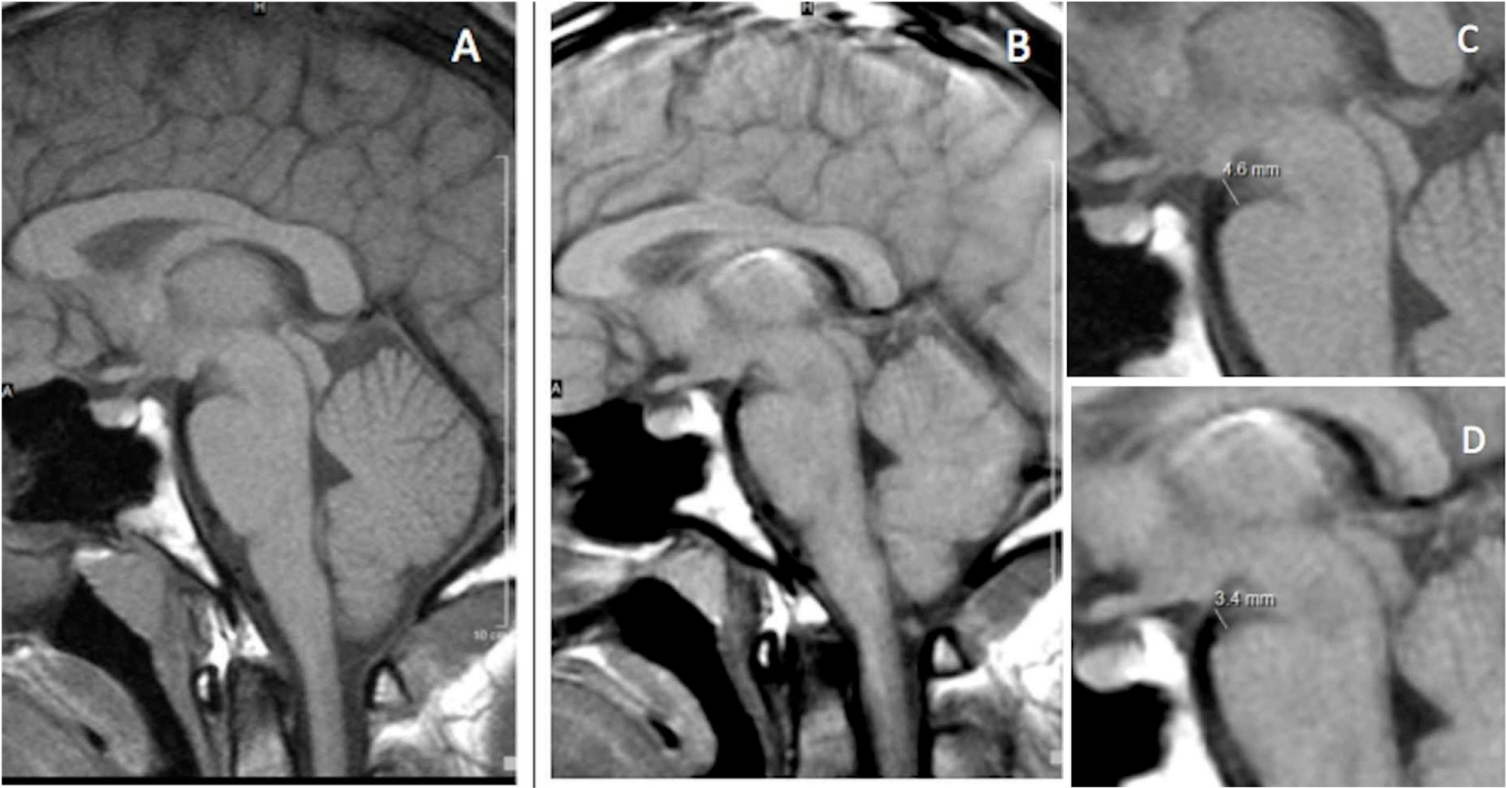
Case 2 MRI progression (A) MRI brain obtained from a concussion that had occurred 13 months prior to current injury, with a measured mamillopontine distance of 4.6 millimeters (highlighted in image C). (B) MRI brain obtained from current concussion showing a decreased mamillopontine distance of 3.4 millimeters (highlighted in image D). The observed decrease of the mamillopontine distance is a finding in intracranial hypotension.

He was reevaluated the next day in the athletic training facility and complained of headache, insomnia, photophobia, irritability, and difficulty concentrating. He described having a “funny feeling” in his head with bending forward at the waist and then returning to an upright position. His medical history was significant for a concussion 13 months prior that resolved over three weeks. A brain MRI obtained at that time was negative for any intracranial pathology (Figure 4A, 4C); however, an MRI obtained at the end of week 3 of this current concussive episode showed signs of intracranial hypotension (Figure 4B, 4D). Personal and family history was negative for headaches. He was taking no medications.

On physical exam, he was in no acute distress, and was alert and oriented to person, place, and time. His cranial nerves 2-12 were intact, and the remainder of his neurological exam, including cerebellar testing with finger-to-nose and heel-to-toe walking, Romberg maneuver, and a subjective simple reaction time maneuver, was normal. Neck exam demonstrated full ROM with mild paraspinal tenderness at C3 level. He had normal strength and sensation in all four extremities. There was mild tenderness to palpation of the bilateral temporomandibular joints (TMJs). Concussion testing was performed and PCSS was >0 in 14 of 22 symptoms with a severity of 42 (prior baseline was >0 for 3 symptoms with a severity of 9), SAC was 23/30 (baseline 23/30), and BESS was 7 (baseline 6). The patient was treated initially with rest, including exclusion from practice, team meetings, and classes. The patient was given acetaminophen for the headaches. His symptoms, including his complaints of headache, improved during the week and on day 6 he performed computerized neurocognitive testing with ImPACT™. His reaction time exceeded the Reliable Change Index compared to his baseline for reaction time composite but his other composite scores did not exceed the Reliable Change Index. 

At the end of week 2, the patient reported he was asymptomatic with normal daily activities. A gradual return to play protocol was attempted; however, he became nauseous while walking quickly on a treadmill and progression was halted. He was seen by a neuropsychologist who felt he had significant fatigue from the concussion as well as vestibular dysfunction. He was referred for vestibular rehabilitation and also started on amantadine 100 mg daily for fatigue related to the injury. He felt much improvement of his fatigue initially on the medication, but the amantadine was discontinued after three days due to nausea and rash. Vestibular rehabilitation did not help his headache. He was then referred to neurology, and an MRI of the brain was obtained.

The MRI, which was performed at the end of week 3, was initially read as normal (Figure 4B) but upon further review and comparison to the previous year’s brain MRI (Figure 4A), intracranial hypotension was suspected when low-lying cerebellar tonsils and decreased mamillopontine distance were recognized (Figure 4B, 4D). The patient then underwent a fluoroscopically guided large volume (15 ml) autologous blood patch at L2/3 level. He reported partial improvement in his activity-related headache symptoms. The patient was placed on caffeine 100 mg three times daily for three days and also on sumatriptan 50 mg as needed. These provided minimal improvement. An MR myelogram cervical/thoracic/lumbar Spine was then performed at the end of week 4 to evaluate for a possible CSF leak, but was negative. Due to his persistent symptoms with activity and a clinical presentation still concerning for intracranial hypotension, a second fluoroscopically guided large volume (15 ml) autologous blood patch at L2/3 level was performed at the end of week 5. Following this second blood patch, the patient's activity-related symptoms improved further but he continued to have intermittent headaches. These remaining headaches were thought to be more consistent with migraines and not related to intracranial hypotension. For the migraines, amitriptyline was started in addition to his sumatriptan.

A repeat brain MRI at eight weeks post injury revealed persistent signs of intracranial hypotension. The patient was not interested in further blood patches at that time. His headache continued to be intermittent but he no longer had positional or activity-related symptoms. By week 13 post injury, he was able to return to full participation in football. The patient was followed closely by team physicians and neurology during the remainder of his collegiate career.

## Discussion

Headache is a common symptom of concussion both acutely and in prolonged concussion symptom settings. Sometimes referred to under the broader term “post-traumatic headache” (PTH), these headaches have a broad array of presentations making treatment decisions challenging. A review in 2006 [3] found that PTH is often characterized by symptoms similar to other primary headache disorders, such as migraine and tension-type, as well as cervicogenic headaches. Also, occipital neuralgia has been implicated in PTH [4]. The preceding two cases illustrate another potential cause of headache in a concussed athlete: intracranial hypotension.

Intracranial hypotension may be related to intracranial hypovolemia, defined as a decreased CSF volume within the cranium, which can occur from under production of CSF or from a loss of CSF referred to as a CSF leak [5]. Intracranial hypovolemia however does not always result in the clinical and imaging features associated with intracranial hypotension, indicating a more complex etiology to intracranial hypotension [6]. Intracranial hypotension is most often secondary to a CSF leak, the cause of which can be spontaneous, traumatic, or iatrogenic [5]. Spontaneous CSF leaks are often due to meningeal diverticula and an underlying connective tissue disorder [2,6]. Iatrogenic CSF leaks often occur in the context of lumbar puncture, spinal surgery, and ear, nose, and throat procedures [2]. Traumatic CSF leaks have been reported with head trauma and are often intracranial in nature [7]. However, spinal CSF leaks have also been reported in 50% of whiplash injuries, mainly in the lumbar and lower thoracic spine, and more specifically in the area of the nerve root sleeves [8]. Whiplash injuries involve rapid hyperextension-hyperflexion movements of the cervical spine, similar to the mechanism of injury in our two cases above (cervical hyperextension). 

One way to describe the association between intracranial pressure and volume is the Monro-Kellie Hypothesis. This hypothesis states that the sum of volumes of brain, CSF, and intracranial blood is constant [9]. A decrease in one should cause an increase in one or both of the remaining mediums. Many MRI abnormalities associated with intracranial hypotension or CSF volume depletion can be explained by this balance [9].

While a positional or orthostatic headache is the most classic symptom of headaches due to spontaneous intracranial hypotension (SIH), other varieties of headache and a myriad of associated symptoms (neck pain, nausea, vomiting, photophobia, phonophobia, auditory and visual changes) have been reported, making diagnosis of SIH difficult by symptoms alone [2,6]. MRI of the brain has emerged as a useful diagnostic tool for SIH, and a constellation of findings may be seen. These findings occur in varying degrees of frequency with pachymeningeal enhancement regarded as the most common finding [10]. Our athletes had engorgement of the venous structures and sagging of the brain; however, neither athlete was found to have pachymeningeal enhancement as their studies were performed without gadolinium contrast. Despite these numerous findings, between 20% and 28% of patients with SIH will have a normal brain MRI [2,10].

Both injuries reported above occurred in athletes playing American football. To our knowledge, no cases of post-concussive headache related to intracranial hypotension sustained during an athletic activity have been reported in the literature. One specific case of trauma-related post-concussive headache as a sequela of intracranial hypotension has been published [11]. This case showed similarities when compared to the two above: young, healthy male individual, improvement in symptoms when lying flat, initial head CT unremarkable, MRI findings (low lying cerebellar tonsils, sagging brainstem). However, the etiology was not revealed as a sport-specific injury. 

In the two cases above, both athletes experienced significant cervical extension during their injuries, which could have caused a CSF leak. Despite this speculation, it is not possible to definitively link the concussive injury with the development of the CSF leak. CT myelogram was not performed at the time of injury, and brain MRIs were not obtained until 7.5 weeks (case 1) and 3 weeks (case 2) after injury. It is possible that both players simply developed spontaneous CSF leaks while recovering from their concussions. Regardless of cause, traumatic versus spontaneous, intracranial hypotension was present during the time of their post-concussion headaches. Symptoms and radiographic abnormalities resolved following treatment with EBPs.

## Conclusions

The cause of prolonged post-concussion headaches is often unknown. Both cases presented above involved collegiate football players who sustained concussions associated with persistent headaches and were subsequently found to have findings suggestive of intracranial hypotension by MRI, consistent with a CSF leak. These cases are clinically significant because intracranial hypotension caused by a CSF leak is a potentially under-recognized cause of post-concussion headache, and may lead to delayed return to school, sport, and work. Both athletes responded favorably to EBP, demonstrated evidence of improvement of intracranial volume on MRI, and successfully returned to normal function. The prevalence of intracranial hypotension following concussion is unknown; however, we believe this entity should be considered in the differential diagnosis for post-concussive headache, especially if there is a postural component to the headache and a history of significant cervical motion. Furthermore, MRI should be considered relatively early in an athlete with a post-concussion headache who is stagnant in their recovery, and is displaying symptoms concerning for intracranial hypotension.
